# Complete Genome Sequence of the *Campylobacter helveticus* Type Strain ATCC 51209

**DOI:** 10.1128/genomeA.00398-17

**Published:** 2017-05-25

**Authors:** William G. Miller, Emma Yee, James L. Bono

**Affiliations:** a Produce Safety and Microbiology Research Unit, Agricultural Research Service, U.S. Department of Agriculture, Albany, California, USA; b Meat Safety and Quality Research Unit, Agricultural Research Service, U.S. Department of Agriculture, Clay Center, Nebraska, USA

## Abstract

*Campylobacter helveticus* has been isolated from domestic dogs and cats. Although *C. helveticus* is closely related to the emerging human pathogen *C. upsaliensis*, no *C. helveticus*-associated cases of human illness have been reported. This study describes the whole-genome sequence of the *C. helveticus* type strain ATCC 51209 (=CCUG 30682^T^).

## GENOME ANNOUNCEMENT

*Campylobacter helveticus* is a catalase-negative, thermotolerant campylobacter that is generally isolated from cats and occasionally from dogs (1–4). However, although it is isolated from pets and is related to the human emerging pathogen *C. upsaliensis* (5), there have been no reports of *C. helveticus*-associated human disease (5, 6). The *C. helveticus* type strain ATCC 51209 was isolated in Switzerland from a domestic cat with diarrheal illness (7). In this study, we report the first closed genome sequence of strain ATCC 51209^T^.

The Roche GS-FLX, Illumina HiSeq, and PacBio RS next-generation sequencing platforms were used to complete the strain ATCC 51209^T^ genome. Shotgun and paired-end Roche 454 reads were assembled (using Newbler version 2.6) into 6 scaffolds (1 chromosomal and 5 plasmid) of 46 contigs interspersed with unique and repeat contigs. Three plasmid scaffolds contained a single contig each and were closed/circularized using HiSeq reads (SeqWright, Houston, TX, USA). Two of the remaining three scaffolds were closed into a single contig using a combination of PCR amplification/Sanger sequencing across the contig gaps and PacBio sequencing. Base calls on all six final contigs were validated using HiSeq reads. The final coverage across the genome was 1,314×. Assembly of the chromosome was further validated using an optical restriction map (restriction enzyme *Nhe*I; OpGen, Gaithersburg, MD, USA).

*C. helveticus* strain ATCC 51209^T^ has a circular genome of 1,759 kb with an average GC content of 34.6%. Protein-, rRNA- and tRNA-encoding genes were identified as previously described (8). The genome contains 1,698 putative protein-coding genes and 75 pseudogenes. Three rRNA operons were identified; however, all three operons are split, where the 23S and 16S ribosomal RNA genes are separated by 21, 353, or 4 kb. Two large genetic islands of 107 and 83 kb were identified in the ATCC 51209^T^ chromosome, as well as three smaller elements of 4, 6, and 10 kb. The chromosome also contains 82 GC tracts ≥8 bp.

Five plasmids were identified in the *C. helveticus* type strain: two large plasmids of 72 (pHELV-1) and 34 (pHELV-2) kb (GC = 30.1%), and three small plasmids of 3.3 (pHELV-3), 1.7 (pHELV-4), and 1.8 (pHELV-5) kb (GC = 30.5 to 32.8%). The pHELV-1 sequence could not be circularized using reads from any of the next-generation platforms. Furthermore, pHELV-1 contains a perfect 606-bp terminal inverted repeat, suggesting that pHELV-1 is a linear plasmid (9). pHELV-3 is a *repAB* family theta-replicating, iteron-containing plasmid (10). pHELV-5 is a RepL family plasmid; however, pHELV-5 RepL is more similar to RepL proteins in *Firmicutes* spp., with no similarity to RepL proteins identified previously in *Campylobacter* spp. (10).

A noteworthy feature of the *C. helveticus* type strain genome is the presence of a large suite of restriction/modification (R/M) genes. The ATCC 51209^T^ genome is predicted to encode seven R/M systems: one type I, four type II, and two type III; two additional type II systems are also encoded, although in each case the R subunit is predicted to be a pseudogene. Besides these systems, 10 DNA methyltransferases, 2 restriction endonucleases, and 10 R/M-associated putative pseudogenes were identified.

### Accession number(s).

The complete genome sequence of *C. helveticus* strain ATCC 51209^T^ has been deposited in GenBank under the accession numbers CP020478 (chromosome) and CP020479 to CP020483 (plasmids pHELV-1 to pHELV-5).
